# Early-Life Wheezing and Atopic Burden Are Associated with Treatment-Defined Asthma Severity and Control in School-Age Children: A Two-Center Cross-Sectional Study

**DOI:** 10.3390/jcm15166459

**Published:** 2026-08-20

**Authors:** Mehmet Şirin Kaya, Asena Pınar Sefer, Sümeyye Baysal, Serdar Göktaş, Burcu Kolukısa

**Affiliations:** 1 Department of Pediatric Immunology and Allergy, Şanlıurfa Training and Research Hospital, Şanlıurfa 63040, Türkiye; drsumeyyebaysal@gmail.com; 2 School of Medicine, Department of Pediatrics, Division of Allergy and Immunology, Recep Tayyip Erdoğan University, Rize 53200, Türkiye; asenapinar.seferarinc@erdogan.edu.tr; 3 Department of Pediatric Immunology and Allergy, SBÜ Van Training and Research Hospital, Van 65000, Türkiye; drsrdr33@gmail.com (S.G.); burcu.kocamis@gmail.com (B.K.)

**Keywords:** childhood asthma, clinical stratification, phenotype, wheezing, atopy, asthma severity, passive smoking

## Abstract

**Background:** Childhood asthma is clinically heterogeneous, yet simple clinical approaches for describing concurrent disease burden are limited. We tested whether a two-axis phenotype matrix—built from early-onset and/or high-burden wheeze and atopic load—is associated cross-sectionally with treatment-defined severity and control in school-aged children with asthma. This exploratory, unvalidated classification requires prospective external validation before clinical use. **Methods:** In a two-center retrospective cross-sectional study, 337 children aged 6–12 years with physician-confirmed asthma were classified into four clinical strata (low-burden, familial/atopic-dominant, wheeze-dominant, atopic-wheezing). The primary outcome was moderate-to-severe asthma, defined by controller treatment step and deliberately independent of acute events. Variables independently associated with the outcomes were identified using multivariable logistic regression with prespecified sensitivity analyses; trends across ordered wheezing-onset categories were assessed using the Cochran–Armitage trend test, adjusted associations were confirmed by likelihood-ratio tests, and secondary outcomes were false-discovery-rate corrected. **Results:** The atopic-wheezing stratum was the largest group (140/337, 41.5%) and carried the highest burden: moderate-to-severe asthma 49.3% vs. 17.4% in low-burden and uncontrolled asthma 42.1% vs. 13.0% (both *q* < 0.01). Adverse outcomes were generally more frequent with earlier wheezing onset (moderate-to-severe asthma 26.9% to 57.9% from no early wheeze to onset ≤ 12 months; *p* for trend <0.001). After adjustment, the atopic-wheezing stratum had the largest adjusted odds ratios for both moderate-to-severe asthma (aOR 4.49, 95% CI 1.91–10.54) and uncontrolled asthma (aOR 4.78, 95% CI 1.85–12.35), and remained significant across all sensitivity analyses. Passive smoke exposure was independently associated with uncontrolled asthma (aOR 1.86, 95% CI 1.13–3.05) but not severity. The FEV1/FVC z-score differed significantly across strata and was lowest in the atopic-wheezing group (median z-score −1.28 vs. +0.10 in low-burden; *p* < 0.001). **Conclusions:** The co-occurrence of high atopic load with early-onset or high-burden wheezing defined the clinical stratum with the highest observed treatment requirement and poorest control, using only routine history and standard allergy work-up. Passive smoke exposure was independently associated with uncontrolled asthma.

## 1. Introduction

Childhood asthma is a heterogeneous clinical condition, with substantial variation in symptom burden, exacerbation frequency, treatment requirements, asthma control, and lung function. This heterogeneity often emerges early in life and is not fully captured by conventional severity labels alone [1,2]. Longitudinal birth-cohort studies have shown that childhood wheeze follows distinct temporal trajectories, including transient early, persistent, intermediate-onset, and late-onset patterns, which are associated with differing probabilities of persistent asthma symptoms, airway hyperresponsiveness, and impaired lung function at school age [3,4,5].

The clinical significance of early-life wheeze depends not only on the age at first episode, but also on recurrence, persistence, and the accompanying allergic profile. Persistent or recurrent wheeze, particularly in children with early allergic sensitization, has been associated with a greater burden of respiratory symptoms and poorer lung function at school age than transient early wheeze [4,5,6]. Although detailed wheeze trajectories provide valuable insight in research settings, such longitudinal information is rarely available in routine practice. Clinical assessment therefore commonly relies on the reported timing of first wheeze, the frequency of wheezing episodes, and the occurrence of wheeze outside respiratory infections.

Allergic disease represents a second important dimension of childhood asthma heterogeneity. The timing, breadth, and pattern of sensitization may be more informative than the presence of any single allergic diagnosis or sensitization result [6,7]. Eczema, allergic rhinitis, food allergy, aeroallergen sensitization, and a family history of asthma or atopy frequently coexist; however, their developmental relationships are heterogeneous and do not necessarily conform to a uniform atopic march [6,7,8]. Accordingly, the aggregation of allergic and familial characteristics may provide more clinically meaningful information than individual features considered in isolation.

Data-driven phenotyping studies further underscore the limitations of describing childhood asthma using a single clinical variable. Latent-class and cluster-based analyses have identified subgroups that differ in age at onset, allergic comorbidity, symptom burden, treatment intensity, airflow limitation, and bronchial responsiveness [1,2,9,10,11]. However, such classifications are generally derived from longitudinal cohorts or multivariable modelling approaches that require extensive phenotyping and may not be readily transferable to routine outpatient care.

By contrast, early wheezing history, wheeze burden, allergic comorbidities, aeroallergen sensitization, and family history are routinely available during pediatric allergy assessment. Related variables are incorporated into prediction tools such as the Asthma Predictive Index and its modified version; however, these tools were developed primarily to estimate the likelihood of future asthma among preschool children with recurrent wheeze, rather than to characterize disease burden in school-aged children with established asthma [12,13].

We therefore evaluated a pragmatic 2 × 2 clinical stratification framework integrating two routinely available domains: early-onset and/or high-burden wheeze, and atopic load. We examined whether the resulting clinical strata differed with respect to treatment-defined asthma severity, asthma control, recent exacerbations, emergency department visits, systemic corticosteroid use, and spirometric indices among school-aged children with physician-confirmed asthma. This framework was intended as a descriptive clinical stratification approach, rather than as a biological endotype or a validated prognostic model. This was a predefined, rule-based stratification and was not derived using latent-class, cluster, or other data-driven methods.

## 2. Materials and Methods

### 2.1. Study Design and Population

This two-center retrospective cross-sectional study was conducted at two tertiary pediatric allergy clinics between March 2023 and October 2024 using routinely maintained medical records. The two centers are tertiary referral clinics serving urban and rural populations in Şanlıurfa (Southeastern Türkiye) and Van (Eastern Türkiye), respectively. The index assessment was defined as the most recent outpatient visit during the study period at which treatment step, asthma control, and the variables required for clinical-stratum assignment could be evaluated concurrently; acute outcomes were assessed over the 12 months preceding this index assessment.

Children were eligible if they were 6–12 years of age, had been followed for at least 12 months, and had asthma confirmed by a pediatric allergy specialist in accordance with Global Initiative for Asthma (GINA) recommendations [14]. Asthma diagnosis was based on a compatible history of variable respiratory symptoms and, where objective testing was available, documented evidence of variable expiratory airflow limitation. Children with cystic fibrosis, bronchiectasis, primary ciliary dyskinesia, bronchopulmonary dysplasia, congenital lung abnormalities, primary immunodeficiency, chronic aspiration syndrome, severe neuromotor impairment, active tuberculosis, clinically relevant pulmonary sequelae of previous tuberculosis, or chronic lung disease other than asthma were excluded. Records lacking the information required for group assignment or outcome assessment were also excluded.

A total of 1860 outpatient records from the two clinics were screened during the study period. Medical-record screening and data abstraction were performed by five physicians experienced in pediatric allergy and immunology using a standardized data-extraction form; uncertain cases were jointly reviewed and resolved by consensus. After removal of duplicate or repeat-visit records (*n* = 486), 1374 unique patient charts were assessed for eligibility. Of these, 1037 were excluded because of age outside the prespecified 6–12-year range (*n* = 321), follow-up shorter than 12 months (*n* = 204), lack of physician-confirmed asthma (*n* = 228), presence of exclusionary chronic pulmonary or systemic disease (*n* = 74), or insufficient information for phenotype assignment and/or outcome assessment (*n* = 210). The final analytic cohort comprised 337 children (Supplementary Figure S1). Spirometry, ACT/C-ACT, and some laboratory variables were not required for cohort inclusion and were analysed on an available-case basis; spirometry was available in 220 of 337 children (65.3%) and ACT/C-ACT in 254 of 337 (75.4%).

The study was approved by the Harran University Clinical Research Ethics Committee (18 November 2024; session no. 18; decision no. HRÜ/24.18.35). Data were extracted from the anonymized records after ethics approval had been obtained. The requirement for informed consent was waived because of the retrospective design and use of de-identified data. The study was conducted in accordance with the Declaration of Helsinki and is reported in accordance with the STROBE statement [15].

### 2.2. Data Collection and Definitions

Demographic, clinical, allergic, and asthma-related data were extracted from medical records. Information on early-life wheeze was obtained primarily from contemporaneous clinical documentation. When such documentation was unavailable, parental report recorded during routine clinical assessment was used. For each child, we recorded whether the early-wheeze history was based on contemporaneous clinical documentation or on parental report; this source was used in a prespecified sensitivity analysis restricted to documented (non-recall) histories. Because parental report of early wheeze may be subject to recall and interpretation error, this is a potential source of misclassification, which we addressed through that sensitivity analysis.

Early-onset wheeze was defined as a first documented or reported wheezing episode at or before 24 months of age. High wheeze burden was defined as at least three wheezing episodes during the first 6 years of life or wheeze occurring apart from respiratory infections. These variables were selected based on previous studies of early-life wheeze trajectories and pediatric asthma prediction models [1,2,3,12,13]. The wheeze domain was considered positive when either criterion was present. Early onset and high wheeze burden were combined using an OR rule because each captures a clinically relevant but partly distinct aspect of early wheezing illness, and either criterion alone may incompletely identify children with an adverse wheezing history in retrospective records.

Atopic dermatitis, allergic rhinitis, and food allergy were recorded when documented by the treating clinician. Aeroallergen sensitization was defined as a positive skin-prick test and/or serum allergen-specific immunoglobulin E (sIgE) result to at least one aeroallergen. Parental asthma and family history of atopy were recorded from the medical record.

High atopic load (encompassing both personal atopic features and a family history of atopy or asthma) was defined as the presence of at least two of the following features: atopic dermatitis, food allergy, allergic rhinitis, aeroallergen sensitization, parental asthma, and family history of atopy. Eosinophilia was defined as an absolute blood eosinophil count of at least 300 cells/µL or a peripheral eosinophil proportion of at least 4%. Polysensitization was defined as sensitization to two or more distinct allergen groups. Passive smoke exposure was defined as documented household exposure to tobacco smoke. Prematurity was recorded when documented in the medical record. The six atopic features were assigned equal weight because the framework was designed as a simple, rule-based clinical stratification using routinely documented binary variables rather than as a fitted prediction score. The threshold of at least two features was prespecified as a pragmatic definition of accumulated atopic burden, and robustness was examined using a stricter threshold of at least three features. Because parental asthma and a family history of atopy may overlap, their individual associations were additionally examined in component-wise sensitivity analyses.

### 2.3. Clinical Stratification

Participants were classified by crossing the wheeze domain with atopic load. Children with low atopic load and no early-onset or high-burden wheeze were assigned to the low-burden group. Those with high atopic load but no early-onset or high-burden wheeze were assigned to the familial/atopic-dominant group. Children with early-onset and/or high-burden wheeze and low atopic load were assigned to the wheeze-dominant group. Children meeting both the wheeze-domain and high-atopic-load criteria were assigned to the atopic-wheezing group. For brevity, the four clinical strata are at times referred to as ‘phenotypes’; the term is used descriptively and does not imply biologically distinct phenotypes or endotypes.

### 2.4. Outcome Measures

The primary outcome was treatment-defined moderate-to-severe asthma. Children were classified as having moderate-to-severe asthma if they were receiving GINA Step 3–5 controller treatment at the recorded clinical assessment or had a chart-documented specialist classification of moderate-to-severe asthma [14]. Acute asthma events were not included in this definition. We use the term ‘treatment-defined’ to emphasize that GINA Step 3–5 denotes the controller treatment required to achieve or maintain control rather than intrinsically severe (difficult-to-treat) asthma, which requires confirmation of adherence and inhaler technique on optimized therapy; the outcome should therefore be interpreted as a marker of treatment requirement and current control. Because this outcome combines two criteria—current GINA Step 3–5 controller treatment or a chart-documented specialist classification of moderate-to-severe asthma—it represents a composite indicator of treatment requirement and management intensity rather than a single objective measure of intrinsic (biological) severity.

Asthma control was assessed separately from asthma severity. Uncontrolled asthma was defined as an Asthma Control Test (ACT) or Childhood Asthma Control Test (C-ACT) score below 20 when questionnaire data were available [16,17]. For children without an available ACT or C-ACT score, the treating specialist’s contemporaneous assessment recorded in the medical record was used. Acute asthma outcomes were assessed during the 12 months preceding the recorded clinical assessment and included exacerbations, emergency department visits, and systemic corticosteroid courses. An exacerbation was defined as an acute worsening of asthma requiring systemic corticosteroid treatment or urgent medical assessment [18].

Spirometric data obtained during routine clinical care were analyzed among children with available measurements. Forced expiratory volume in 1 s (FEV1), forced vital capacity (FVC), and the FEV1/FVC ratio were recorded and interpreted using the Global Lung Function Initiative (GLI-2012) reference equations [19]. For each child, the measured FEV1/FVC ratio was converted to a z-score based on sex, age, and standing height using the GLI-2012 Caucasian reference module, and airflow limitation was defined as an FEV1/FVC below the lower limit of normal (LLN; z-score < −1.645, corresponding to the 5th percentile). FEV1/FVC is reported as a z-score in the primary analysis; a fixed FEV1/FVC ratio below 80% is reported only as a secondary sensitivity threshold to preserve comparability with earlier literature. Bronchodilator reversibility was defined as an increase in FEV1 of at least 12% from baseline after salbutamol administration. Spirometry had been performed as part of routine clinical care according to local laboratory protocols; because the data were collected retrospectively, formal central re-grading of maneuver acceptability and repeatability was not undertaken.

### 2.5. Statistical Analysis

**Figure 1:**
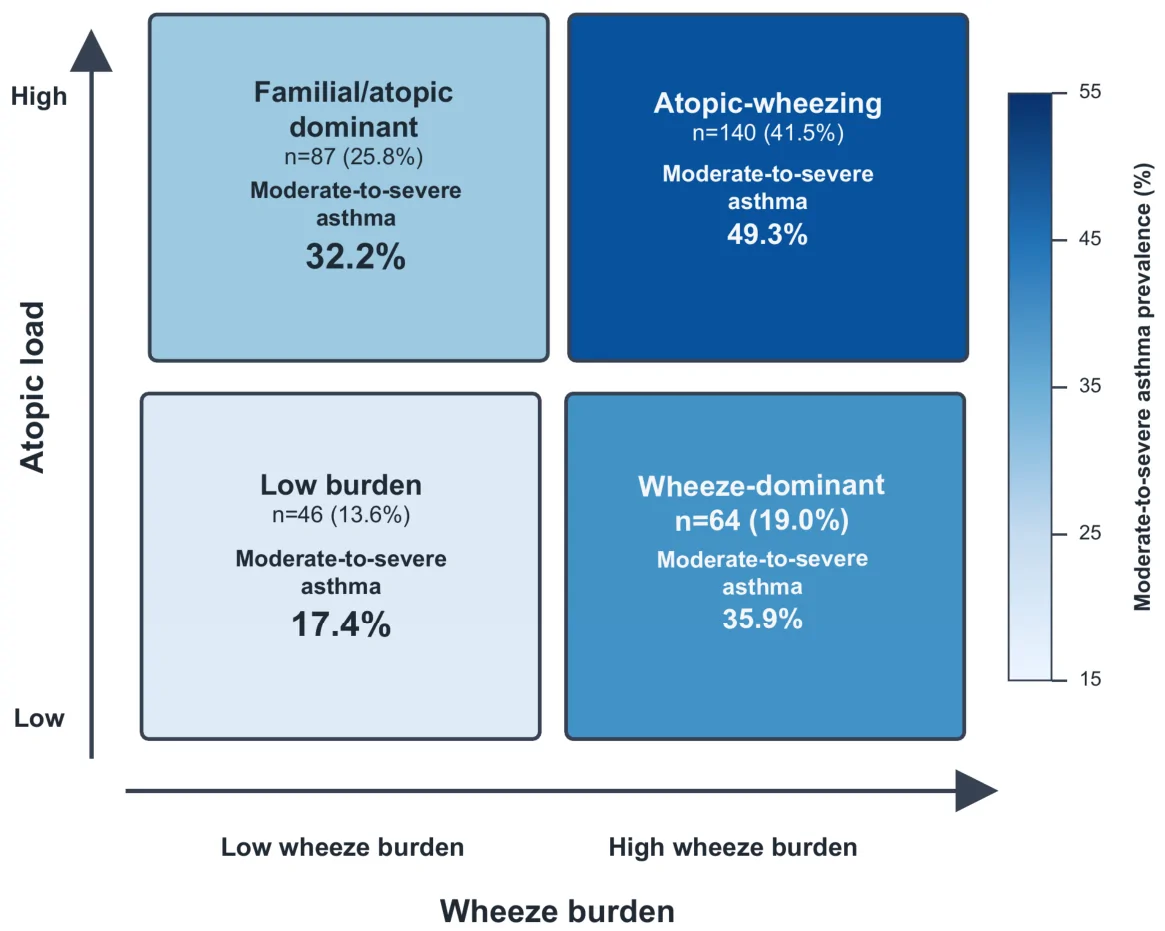
Clinical stratification matrix and within-stratum prevalence of moderate-to-severe asthma. Children were classified by crossing wheeze burden (horizontal axis) with atopic load (vertical axis). Each cell displays the stratum label, number and proportion of children, and the within-stratum prevalence of moderate-to-severe asthma. Cell shading corresponds to the prevalence of moderate-to-severe asthma, with darker blue indicating a higher prevalence. Clinical outcomes were not used to assign stratum membership.

**Figure 2:**
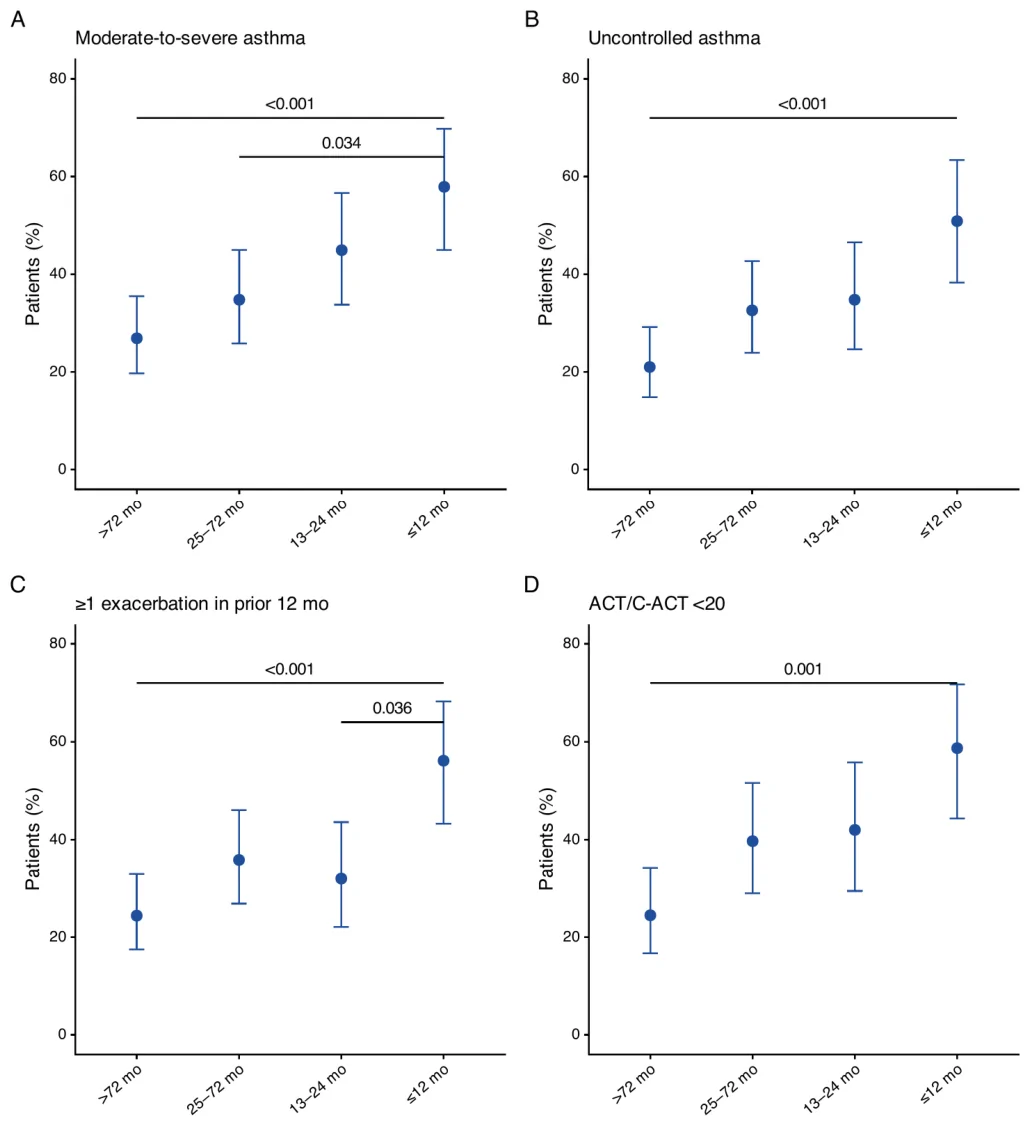
Adverse asthma outcomes according to age at first wheezing episode. (**A**) Prevalence of moderate-to-severe asthma. (**B**) Prevalence of uncontrolled asthma. (**C**) Prevalence of at least one exacerbation during the preceding 12 months. (**D**) Prevalence of poor asthma control, defined as an ACT/C-ACT score < 20. Points represent unadjusted proportions, and whiskers indicate 95% Wilson confidence intervals. Overall differences across the four age-at-first-wheezing categories were assessed using Pearson chi-square tests. Brackets indicate statistically significant pairwise comparisons based on two-sided Fisher exact tests with Holm adjustment for six pairwise comparisons within each outcome; only statistically significant comparisons are displayed. Group sizes were 119, 92, 69, and 57 for the no-early-wheeze, 25–72-month, 13–24-month, and ≤12-month groups, respectively. Analysis in panel (**D**) was restricted to children with available ACT/C-ACT scores; group denominators were 90, 68, 50, and 46, respectively. ACT, Asthma Control Test; C-ACT, Childhood Asthma Control Test; CI, confidence interval; mo, months.

**Figure 3:**
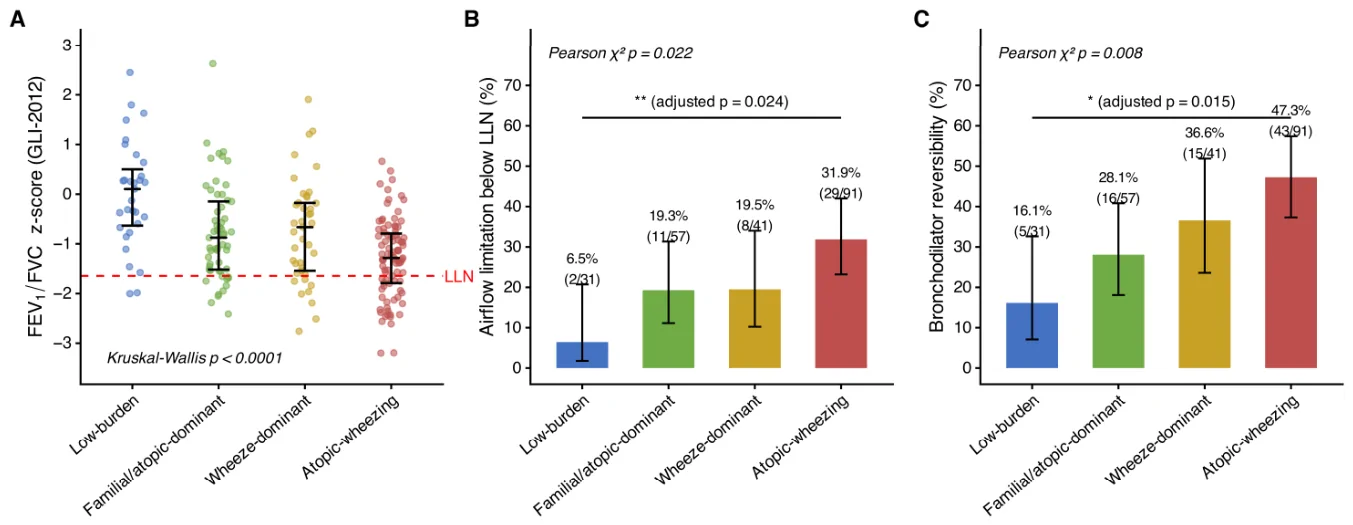
Pulmonary function measures according to clinical strata. (**A**) FEV_1_/FVC z-scores (GLI-2012) across the four clinical strata. Individual points represent children; horizontal bars indicate medians and interquartile ranges. FEV_1_/FVC z-scores differed across strata (Kruskal–Wallis *p* < 0.0001). Pairwise comparisons were performed using Dunn’s multiple-comparisons test. (**B**) Prevalence of airflow limitation, defined as FEV_1_/FVC below the lower limit of normal (GLI-2012 z-score < −1.645), according to stratum. Overall differences were assessed using the Pearson chi-square test (*p* = 0.022); displayed pairwise comparisons were assessed using two-sided Fisher’s exact tests with Holm adjustment. (**C**) Prevalence of bronchodilator reversibility according to stratum. Overall differences were assessed using the Pearson chi-square test (*p* = 0.008); the displayed pairwise comparison was assessed using a two-sided Fisher’s exact test with Holm adjustment. Asterisks above brackets indicate adjusted pairwise *p* values: * *p* < 0.05 and ** *p* < 0.01. In panel (A), the red dashed line indicates the GLI-2012 lower limit of normal (z-score −1.645); colors identify the four clinical strata consistently across panels. Error bars in panels (**B**,**C**) indicate 95% Wilson confidence intervals. Analyses were restricted to children with available spirometry data (*n* = 220). FEV_1_, forced expiratory volume in 1 s; FVC, forced vital capacity; IQR, interquartile range.

Statistical analyses were performed using jamovi version 2.7 (The jamovi Project, Sydney, Australia). Figure 1 was prepared using jamovi, and Figure 2 and Figure 3 were prepared using R version 4.6.0 (R Foundation for Statistical Computing, Vienna, Austria) within RStudio version 2025.05.1+513 (Posit Software, PBC, Boston, MA, USA).

Continuous variables were summarized as mean ± standard deviation (SD) or median (interquartile range [IQR]), as appropriate. Categorical variables were summarized as frequencies and percentages. Distributional assumptions were assessed using the Shapiro–Wilk test and visual inspection of graphical displays. Group comparisons were performed using one-way analysis of variance, the Kruskal–Wallis test, Pearson’s chi-square test, or Fisher’s exact test, as appropriate. Trends in binary outcomes across the ordered wheezing-onset categories (no early wheeze, 25–72, 13–24, and ≤12 months) were assessed using the Cochran–Armitage trend test. Proportions were presented with 95% Wilson confidence intervals where relevant.

Associations between clinical exposures and binary outcomes were evaluated using multivariable logistic regression. Results are reported as odds ratios (ORs) or adjusted odds ratios (aORs) with 95% confidence intervals (CIs). Categorical predictors were modeled using indicator variables with prespecified clinically relevant reference categories. Covariates were selected a priori based on clinical relevance and included demographic, environmental, familial, atopic, inflammatory, and center-related factors, as appropriate. Overall associations with multi-level categorical predictors were evaluated using likelihood ratio tests comparing nested models. Effect modification was assessed by including multiplicative interaction terms in the corresponding regression models. Because parental asthma and the other atopic features contribute to the phenotype definition, they were not re-entered alongside the phenotype variable; their individual contributions were instead examined in separate exploratory single-predictor models, and a two-axis model entering the wheeze domain (early-onset and/or high-burden wheeze) and high atopic load separately, together with their multiplicative interaction term, was used to test whether the two axes acted additively or synergistically. Additive interaction on the risk scale was assessed using adjusted risk ratios estimated from a modified Poisson regression model with robust (sandwich) variance, from which the relative excess risk due to interaction (RERI), the attributable proportion, and the synergy index were derived with bootstrap 95% confidence intervals. For the primary model, discrimination (c-statistic) and calibration (calibration slope and the Hosmer–Lemeshow test) were assessed, and optimism-corrected estimates were obtained by bootstrap resampling with 1000 replications. This internal bootstrap procedure assessed the stability and optimism of the multivariable regression model only and does not constitute validation of the 2 × 2 clinical stratification itself, which remains to be externally validated.

Where post hoc pairwise comparisons were conducted following statistically significant overall tests, *p*-values were adjusted using the Holm procedure. Sensitivity analyses were conducted using alternative model specifications, exposure definitions, analytic restrictions, and subgroup analyses. These analyses were considered exploratory and interpreted accordingly.

Analyses were conducted using available-case data, and no imputation was performed. All tests were two-sided, and *p* values < 0.05 were considered statistically significant, except where multiplicity-adjusted *p* values were reported.

## 3. Results

### 3.1. Study Population and Clinical Strata

A total of 337 children were included in the analysis. The mean age was 9.2 ± 1.4 years and 192 participants (57.0%) were male. Documented prematurity was present in 36 children (10.7%), with an equal sex distribution (18 boys and 18 girls). Previous bronchiolitis hospitalization (*n* = 52) and early-onset wheeze overlapped in 25 children; additional adjustment for bronchiolitis hospitalization did not materially alter the association between the atopic-wheezing stratum and moderate-to-severe asthma. Participants were recruited from Center 1 (*n* = 182, 54.0%) and Center 2 (*n* = 155, 46.0%). Center-specific data are shown in Supplementary Table S5.

Early-onset wheezing was reported in 126 children (37.4%). High wheeze burden was present in 204 children (60.5%), and 227 children (67.4%) met the predefined criteria for high atopic load. Allergic rhinitis and aeroallergen sensitization were the most prevalent atopic features, occurring in 216 children (64.1%) and 235 children (69.7%), respectively. Overall, 128 children (38.0%) had moderate-to-severe asthma, and 108 children (32.0%) had uncontrolled asthma. Spirometry data were available for 220 participants (65.3%), while ACT/C-ACT data were available for 254 participants (75.4%). Baseline demographic, clinical, and atopic characteristics are summarized in Table 1. Availability of spirometry and ACT/C-ACT was broadly similar across the four clinical strata, ranging from approximately 64% to 67% and 73% to 76%, respectively. Comparisons of children with and without available spirometry and ACT/C-ACT data are presented in Supplementary Table S6.

The rule-based classification assigned 46 children (13.6%) as low-burden, 87 (25.8%) as familial/atopic-dominant, 64 (19.0%) as wheeze-dominant, and 140 (41.5%) as atopic-wheezing. The prevalence of moderate-to-severe asthma was lowest in the low-burden phenotype (17.4%) and highest in the atopic-wheezing phenotype (49.3%) (Figure 1). Detailed clinical and atopic characteristics across phenotype groups are presented in Supplementary Table S1.

### 3.2. Age at First Wheeze and Asthma Outcomes

Among 337 children, the age-at-first-wheeze groups comprised no early wheeze (first wheeze > 72 months; *n* = 119), 25–72 months (*n* = 92), 13–24 months (*n* = 69), and ≤12 months (*n* = 57). The prevalence of adverse asthma outcomes differed across these categories. Moderate-to-severe asthma was observed in 26.9%, 34.8%, 44.9%, and 57.9% of children with no early wheeze, first wheezing at 25–72 months, 13–24 months, and ≤12 months, respectively (Pearson χ^2^ = 17.62, *p* < 0.001) (Figure 2A). Corresponding prevalences of uncontrolled asthma were 21.0%, 32.6%, 34.8%, and 50.9% (χ^2^ = 16.19, *p* = 0.001) (Figure 2B); ≥1 exacerbation during the preceding 12 months, 24.4%, 35.9%, 31.9%, and 56.1% (χ^2^ = 17.52, *p* < 0.001) (Figure 2C); and ACT/C-ACT < 20, 24.4%, 39.7%, 42.0%, and 58.7%, respectively, among children with available ACT/C-ACT data (χ^2^ = 15.77, *p* = 0.001) (Figure 2D). Across all four outcomes, prevalence generally increased with earlier wheezing onset, although not strictly monotonically for every outcome (for example, exacerbations were 35.9% at 25–72 months and 31.9% at 13–24 months) (Cochran–Armitage *p* for trend < 0.001 for each). In Holm-adjusted pairwise analyses, children with wheezing onset at ≤12 months had significantly higher prevalence of all adverse outcomes than children with no early wheeze (all *p* < 0.001). Moderate-to-severe asthma was also more frequent in the ≤12-month group than in the 25–72-month group (57.9% vs. 34.8%, *p* = 0.034), whereas ≥1 exacerbation was more frequent in the ≤12-month group than in the 13–24-month group (56.1% vs. 31.9%, *p* = 0.036). All remaining pairwise comparisons were not statistically significant after Holm correction.

These associations remained significant after adjustment for current age, sex, study centre, passive smoke exposure, parental asthma, aeroallergen sensitization, and eosinophilia. Compared with no early wheeze, wheezing onset at ≤12 months was associated with higher odds of moderate-to-severe asthma (aOR 3.31, 95% CI 1.67–6.58), uncontrolled asthma (aOR 3.60, 95% CI 1.75–7.38), ≥1 exacerbation during the preceding 12 months (aOR 3.86, 95% CI 1.92–7.78), and ACT/C-ACT < 20 (aOR 4.23, 95% CI 1.90–9.43; all Holm-adjusted *p* ≤ 0.002). Global adjusted associations between wheezing-onset category and each outcome remained significant in likelihood-ratio tests (all *p* ≤ 0.004; Supplementary Table S2).

### 3.3. Stratum-Specific Clinical Outcomes and Type 2-Related Characteristics

Type 2-related characteristics (blood eosinophilia and polysensitization) were examined as type 2-associated features, that is, indirect clinical and laboratory markers commonly associated with type 2 immunity rather than direct measures of airway inflammation. Eosinophilia and polysensitization differed significantly across the four phenotypes. Eosinophilia was observed in 13.0% of children in the low-burden phenotype and 46.4% of those in the atopic-wheezing phenotype (*p* < 0.001). Similarly, polysensitization was present in 8.7% and 59.3% of these groups, respectively (*p* < 0.001) (Table 2).

As expected from the phenotype definition, phenotypes characterized by high atopic load had a greater prevalence of atopic dermatitis, food allergy, allergic rhinitis, aeroallergen sensitization, parental asthma, and family history of atopy than phenotypes with low atopic load (Supplementary Table S1).

Clinical outcomes differed across the four clinical strata, with the lowest burden in the low-burden stratum and the highest in the atopic-wheezing stratum for every outcome (moderate-to-severe asthma 17.4% to 49.3%, *p* < 0.001; uncontrolled asthma 13.0% to 42.1%, *p* = 0.002; full group-specific values are given in Table 2). The prevalence of ≥1 exacerbation, ≥1 emergency department visit, and ≥1 systemic corticosteroid course during the preceding 12 months showed the same pattern, being lowest in the low-burden stratum and highest in the atopic-wheezing stratum. All five prespecified clinical-outcome comparisons remained statistically significant after Benjamini–Hochberg false-discovery-rate correction (all *q* ≤ 0.01) (Table 2).

### 3.4. Pulmonary Function and Bronchodilator Reversibility

Among the 220 children with available spirometry, FEV_1_/FVC z-scores differed across the four clinical strata (Kruskal–Wallis *p* < 0.0001; Figure 3A), being highest in the low-burden stratum (median z-score +0.10) and lowest in the atopic-wheezing stratum (−1.28). In Dunn-adjusted pairwise comparisons the low-burden stratum differed from each of the other three strata, and the atopic-wheezing stratum differed from every other stratum (all adjusted *p* ≤ 0.020), whereas the familial/atopic-dominant and wheeze-dominant strata did not differ (adjusted *p* = 0.83). Notably, the low-burden stratum was centred at a z-score of approximately zero, consistent with airflow near the expected reference mean after standardisation for age, height, and sex. Median (IQR) values for all strata are presented in Table 2.

Airflow limitation, defined as an FEV_1_/FVC below the GLI-2012 lower limit of normal (z-score < −1.645), also differed across strata (Pearson χ^2^, *p* = 0.022; Figure 3B), rising from 2/31 children (6.5%) in the low-burden to 29/91 (31.9%) in the atopic-wheezing stratum; this contrast remained significant after multiplicity adjustment (odds ratio 6.8; adjusted *p* = 0.024), whereas no other pairwise comparison did. In a secondary sensitivity analysis using the previously applied fixed threshold (FEV_1_/FVC < 80%), more children were classified as abnormal (82/220, 37.3%, vs. 50/220, 22.7% by the LLN); the two definitions were concordant in 85.5% of cases, and all discordant children were reclassified from abnormal to normal under the age-appropriate LLN. Stratum-specific values are presented in Table 2.

Bronchodilator reversibility showed a similar pattern (Pearson χ^2^, *p* = 0.008; Figure 3C), increasing from 5/31 children (16.1%) in the low-burden to 43/91 (47.3%) in the atopic-wheezing stratum; only this contrast remained significant after adjustment for multiple comparisons (adjusted *p* = 0.015). Stratum-specific values are presented in Table 2.

### 3.5. Clinical Relevance of the Clinical Strata

In multivariable logistic regression analyses adjusted for age, sex, study center, passive smoke exposure, prematurity, and eosinophilia, the atopic-wheezing phenotype was associated with higher odds of moderate-to-severe asthma compared with the low-burden phenotype (adjusted odds ratio [aOR], 4.49; 95% CI, 1.91–10.54; *p* = 0.001). The atopic-wheezing phenotype was also associated with uncontrolled asthma (aOR, 4.78; 95% CI, 1.85–12.35; *p* = 0.001). The familial/atopic-dominant and wheeze-dominant phenotypes showed intermediate effect estimates. Passive smoke exposure was associated with uncontrolled asthma (aOR, 1.86; 95% CI, 1.13–3.05; *p* = 0.014), but not with moderate-to-severe asthma (Table 3).

The association between the atopic-wheezing phenotype and moderate-to-severe asthma remained statistically significant across all sensitivity analyses. The association persisted after exclusion of eosinophilia from the model (aOR, 4.56; 95% CI, 1.97–10.56), after additional adjustment for parental asthma (aOR, 4.44; 95% CI, 1.86–10.57), and after exclusion of children whose early wheeze history was based solely on parental recall (aOR, 7.55; 95% CI, 2.82–20.17). Center-stratified estimates were directionally consistent and remained statistically significant in both participating centers (Supplementary Table S3).

### 3.6. Additivity of the Two Axes and Component-Wise Contributions

In a two-axis model matching the phenotype definition—crossing the wheeze domain (early-onset and/or high-burden wheeze) with high atopic load—neither the multiplicative interaction (interaction OR 0.80, 95% CI 0.27–2.36; *p* = 0.69) nor the additive interaction on the risk scale (RERI −0.06, 95% CI −2.26 to 0.92; attributable proportion −0.02) was statistically significant, and the wheeze domain and high atopic load were each independently associated with higher odds of moderate-to-severe asthma (OR 2.56, 95% CI 1.01–6.46 and OR 2.24, 95% CI 0.92–5.45, respectively), with no evidence of interaction on either scale; these interaction analyses, however, had limited precision owing to the small doubly unexposed reference stratum. Among the 227 children with a high atopic load, the addition of early-onset or high-burden wheeze (atopic-wheezing versus familial/atopic-dominant phenotype) was associated with approximately two-fold higher adjusted odds of both moderate-to-severe asthma (OR 2.05, 95% CI 1.15–3.66) and uncontrolled asthma (OR 1.98, 95% CI 1.07–3.64). Parental asthma was not independently associated with either moderate-to-severe asthma (OR 1.30, 95% CI 0.80–2.12) or uncontrolled asthma (OR 0.65, 95% CI 0.38–1.11). In an exploratory component-wise analysis, individual atopic features carried only modest associations with moderate-to-severe asthma—reaching significance for atopic dermatitis (OR 1.64, 95% CI 1.02–2.62) and aeroallergen sensitization (OR 1.80, 95% CI 1.08–3.00) but not for food allergy, allergic rhinitis, parental asthma, or family atopy—whereas early-onset and high-burden wheeze showed the largest odds ratios among the individual components examined (OR 2.31, 95% CI 1.45–3.69 and OR 2.12, 95% CI 1.31–3.45) (Supplementary Table S4).

## 4. Discussion

In this two-center real-world cohort of 337 school-aged children with asthma from two under-studied regions of Türkiye, a simple two-axis phenotype matrix effectively distinguished strata with differing concurrent clinical burden. The atopic-wheezing phenotype—defined by the co-occurrence of high atopic load with early-onset or high-burden wheezing—was both the largest group (41.5%) and the stratum with the highest observed concurrent disease burden, showing the highest observed treatment requirement, the poorest current control, the highest observed frequencies of exacerbations and systemic corticosteroid courses, and the lowest airflow. After adjustment it showed the largest adjusted odds ratios for both moderate-to-severe asthma (adjusted OR 4.49, 95% CI 1.91–10.54) and uncontrolled asthma (adjusted OR 4.78, 95% CI 1.85–12.35), and this effect was consistent across all sensitivity analyses. Crucially, the classification draws only on routine history and a standard allergy work-up.

Our finding that earlier wheezing onset was associated with greater treatment-defined severity at school age extends trajectory-based evidence to a routine clinical population. The seminal Tucson work first showed that early wheezers are not a homogeneous group [1], and subsequent birth cohorts demonstrated that early-onset persistent wheeze, particularly when accompanied by allergic sensitization, predicts impaired lung function and greater airway responsiveness at school age [2,3]. In our cohort, moderate-to-severe asthma was more than twice as common among children whose wheezing began at ≤12 months (57.9%) as among those without prominent early wheeze (26.9%), with a significant graded increase across onset categories (*p* for trend < 0.001) that persisted after multivariable adjustment (≤12-month adjusted OR 3.31, 95% CI 1.67–6.58; Supplementary Table S2). Because we used broad onset categories rather than precise months, this signal is unlikely to be an artifact of recall precision; indeed, it did not merely persist but strengthened when the analysis was restricted to documented, non-recall early-wheeze histories (adjusted OR 7.55, 95% CI 2.82–20.17; Supplementary Table S3)—which makes differential recall an unlikely sole explanation, although residual misclassification and the influence of the small restricted sample cannot be excluded.

The second axis, atopic load, was assessed cross-sectionally and reflects the concurrent co-occurrence of atopic features rather than a developmental sequence; the pattern is compatible with, but does not establish, the atopic-march framework [6,7] and with evidence that the multiplicity and pattern of sensitization—rather than the presence of any single comorbidity—track most closely with asthma persistence [5]; detailed birth-cohort modeling further indicates that this progression is not a single fixed sequence but a set of distinct developmental profiles at the individual level [8]. Children accumulating several atopic features clustered in the stratum with the highest observed clinical burden, supporting the view that allergic burden should be counted, not merely noted. Importantly, a high atopic load alone did not define the highest observed clinical-burden group: among children who already carried a high atopic load, the addition of an adverse wheezing history—that is, moving from the familial/atopic-dominant to the atopic-wheezing phenotype—was associated with approximately two-fold higher adjusted odds of both moderate-to-severe asthma (OR 2.05, 95% CI 1.15–3.66; 49.3% vs. 32.2%) and uncontrolled asthma (OR 1.98, 95% CI 1.07–3.64; 42.1% vs. 26.4%). We emphasize that the aim was neither to define a new biological endotype nor to reproduce the Asthma Predictive Index—which forecasts asthma inception in undiagnosed preschoolers [12,13]—but to stratify severity and control among children with established asthma using variables already gathered in routine care.

The ordering of the four clinical strata was corroborated by objective, treatment-independent markers. The FEV_1_/FVC z-score differed across the four strata and was lowest in the atopic-wheezing group (median z-score −1.28 vs. +0.10 in low-burden; Kruskal–Wallis *p* < 0.001), which also had the highest prevalence of airflow limitation below the age-appropriate lower limit of normal and of bronchodilator reversibility. Because lung function was used in neither the phenotype nor the severity definitions, this airflow gradient is an independent corroboration rather than a built-in consequence. Eosinophilia (46.4% vs. 13.0%) and polysensitization (59.3% vs. 8.7%) likewise peaked in the atopic-wheezing group, consistent with a greater burden of type 2–associated features in this stratum, although these cross-sectional markers cannot establish an underlying type 2 endotype [20,21]. Although phenotype-guided, individualized treatment strategies have been investigated in children with asthma [22], the present stratification is descriptive and was not designed to direct therapy. That the five prespecified clinical associations survived false-discovery-rate correction, and that the phenotype effect held across a stricter ≥3-feature atopic-load definition, exclusion of recall-only wheeze data, and center-stratified models (Supplementary Table S3), strengthens confidence that they reflect an observed association gradient rather than chance or a definitional artifact.

Passive smoke exposure was independently associated with poorer asthma control (adjusted OR 1.86, 95% CI 1.13–3.05) but not with treatment-defined severity—a separation that is clinically informative: environmental tobacco smoke may be associated with poorer day-to-day symptom control without necessarily shifting the underlying treatment step or controller requirement. This highlights indoor air quality as a potentially modifiable factor to address at every encounter, particularly in settings where household smoking remains prevalent [23,24]. Parental asthma, by contrast, was not independently associated with either outcome; its association with uncontrolled asthma, although directionally inverse (OR 0.65, 95% CI 0.38–1.11), did not reach statistical significance and should not be over-interpreted.

Why combine these two axes into a matrix rather than a single score? Each axis is independently informative, and crossing them isolates the subgroup exposed to both risk dimensions simultaneously—the atopic-wheezing stratum—that is, the co-occurrence of both clinical domains. Formally, in a model that crossed the wheeze domain (early-onset and/or high-burden wheeze) with high atopic load—mirroring the phenotype definition—the multiplicative interaction was not significant (interaction OR 0.80, 95% CI 0.27–2.36; *p* = 0.69), while each axis was associated with higher odds of moderate-to-severe asthma, with no significant additive interaction on the risk scale either (RERI −0.06, 95% CI −2.26 to 0.92); this indicates no detectable interaction on either scale, although these analyses had limited precision owing to the small reference stratum. Consistent with this, a composite exposure requiring high wheeze burden, high atopic load, and early onset remained independently associated with moderate-to-severe asthma in a separate model (adjusted OR 2.20, 95% CI 1.34–3.59; Supplementary Table S3). A 2 × 2 matrix is thus a transparent way to combine two routinely available and independently informative clinical dimensions without overfitting, flagging the children in whom an atopic predisposition has already been joined by an adverse wheezing history. A component-wise decomposition reinforced this logic: individual atopic features contributed only modestly, and although atopic dermatitis and aeroallergen sensitization each reached significance, no single marker approached the discriminating power of the combined phenotype, while early-onset and high-burden wheeze showed larger associations than the individual atopic features examined (Supplementary Table S4). Because risk is distributed across several correlated, individually modest features—none of which alone matches the adjusted odds ratio of the combined phenotype—it is best captured by combining them rather than relying on any one. Whether such clinically defined childhood phenotypes correspond to stable biological entities remains debated [25,26]; accordingly, our framework is offered as a pragmatic clinical stratification tool rather than a claim about underlying endotypes.

These observations sit within a broader understanding that asthma trajectory and severity are shaped by early-life and environmental exposures, with consequences that can extend into adulthood [27,28,29,30,31]. The high prevalence of early wheezing and atopic burden in this cohort is consistent with a population already on a steeper trajectory and underscores the value of identifying such children early. The use of two geographically and demographically distinct centers in Eastern and Southeastern Anatolia—regions for which phenotyping data are scarce—adds regional relevance, and the consistency of the ordering of the four clinical strata across both centers, with no evidence of a phenotype-by-center interaction (likelihood-ratio test, 3 df, *p* = 0.635; Supplementary Table S3), argues against a single-site idiosyncrasy. This cross-center agreement provides a degree of internal replication but does not substitute for external validation in an independent cohort.

Several limitations warrant emphasis. First, the retrospective design carries potential recall bias for early-childhood wheezing; we mitigated this through broad onset categories and a sensitivity analysis restricted to documented, non-recall histories, in which the phenotype association strengthened rather than weakened, though recall bias cannot be entirely excluded. Second, although the low-burden reference group was clinically meaningful, it was small (*n* = 46, with only eight moderate-to-severe cases), so the phenotype odds ratios—while consistently large and significant—have wide confidence intervals and are best read as estimates of direction and approximate magnitude rather than precise effect sizes. Third, residual confounding from unmeasured factors—including inhaler adherence and technique, socioeconomic status, medication access, obesity, and specific environmental exposures beyond passive smoke—cannot be excluded and may be especially relevant to the passive-smoke association with control. In addition, the ACT and C-ACT are validated but patient- or parent-reported instruments that may not fully reflect objective disease activity; because asthma control was ascertained using these questionnaires when available and the treating physician’s contemporaneous assessment otherwise, some misclassification of asthma control may have been introduced. Fourth, severity and control were assessed cross-sectionally rather than tracked longitudinally, and the absence of a non-asthmatic comparison group precludes inferences about phenotype-associated asthma development. Finally, spirometry and ACT/C-ACT were obtained in routine care and were not available for all participants, limiting those sub-analyses. Most importantly, the matrix was both derived and evaluated within this single cohort; although its performance was consistent across two centers, it has not been tested in an independent cohort and should be regarded at this stage as an exploratory, hypothesis-generating framework.

## 5. Conclusions

In school-aged children with asthma, the co-occurrence of high atopic load with early-onset or high-burden wheezing—the atopic-wheezing stratum—was cross-sectionally associated with a higher treatment requirement, poorer asthma control, and lower lung function, using only routinely available clinical information and standard allergy assessment. The phenotype association was consistent across all sensitivity analyses, and the classification requires no complex algorithm, suggesting potential utility as a simple clinical stratification approach, pending prospective external validation before any routine clinical use. Passive smoke exposure was independently associated with uncontrolled asthma and represents a potentially modifiable exposure that can be addressed at every clinical encounter. Because the matrix was derived and tested within a single cohort, prospective multicenter validation incorporating environmental and inflammatory-biomarker data is warranted before routine clinical adoption.

## Figures and Tables

**Table 1:** Baseline characteristics and outcome prevalence of the study population (*n* = 337).

Characteristic	Value
**Demographic characteristics**	
Age, mean ± SD, years	9.2 ± 1.4
Male sex, *n* (%)	192 (57.0)
Study center, *n* (%)	
Center 1	182 (54.0)
Center 2	155 (46.0)
**Clinical history**	
Prematurity, *n* (%)	36 (10.7)
Bronchiolitis hospitalization, *n* (%)	52 (15.4)
Early-onset wheezing (≤24 mo), *n* (%)	126 (37.4)
High wheeze burden, *n* (%)	204 (60.5)
**Atopic characteristics**	
High atopic load, *n* (%)	227 (67.4)
Atopic dermatitis, *n* (%)	115 (34.1)
Food allergy, *n* (%)	63 (18.7)
Allergic rhinitis, *n* (%)	216 (64.1)
Aeroallergen sensitization, *n* (%)	235 (69.7)
Parental asthma, *n* (%)	98 (29.1)
Family history of atopy, *n* (%)	188 (55.8)
Eosinophilia, *n* (%)	117 (34.7)
Polysensitization, *n* (%)	133 (39.5)
**Environmental exposure**	
Passive smoke exposure, *n* (%)	115 (34.1)
**Asthma outcomes**	
Moderate-to-severe asthma, *n* (%)	128 (38.0)
Uncontrolled asthma, *n* (%)	108 (32.0)

Data are presented as *n* (%) unless otherwise stated. Percentages were calculated using the full study population as the denominator. High wheeze burden and high atopic load were defined according to the prespecified phenotype algorithm. Eosinophilia was defined using the prespecified peripheral blood eosinophil threshold. Polysensitization was defined as sensitization to ≥2 aeroallergen sources.

**Table 2:** Clinical outcomes, Type 2–related characteristics, and pulmonary function measures according to clinical strata.

Variable	Low-Burden (*n* = 46)	Familial/Atopic-Dominant (*n* = 87)	Wheeze-Dominant (*n* = 64)	Atopic-Wheezing (*n* = 140)	*p* Value
**Clinical outcomes**					
Moderate-to-severe asthma, *n* (%)	8 (17.4)	28 (32.2)	23 (35.9)	69 (49.3)	<0.001
Uncontrolled asthma, *n* (%)	6 (13.0)	23 (26.4)	20 (31.2)	59 (42.1)	0.002
≥1 exacerbation, past 12 months, *n* (%)	7 (15.2)	26 (29.9)	23 (35.9)	60 (42.9)	0.005
≥1 ED visit, past 12 months, *n* (%)	4 (8.7)	18 (20.7)	16 (25.0)	46 (32.9)	0.007
≥1 systemic corticosteroid course, past 12 months, *n* (%)	3 (6.5)	16 (18.4)	15 (23.4)	42 (30.0)	0.007
**Type 2-related characteristics**					
Eosinophilia, *n* (%)	6 (13.0)	27 (31.0)	19 (29.7)	65 (46.4)	<0.001
Polysensitization, *n* (%)	4 (8.7)	33 (37.9)	13 (20.3)	83 (59.3)	<0.001
**Pulmonary function measures** *					
FEV_1_/FVC z-score (GLI-2012), median	+0.10	−0.87	−0.66	−1.28	<0.001
Airflow limitation (FEV_1_/FVC < LLN), *n*/*N* (%)	2/31 (6.5)	11/57 (19.3)	8/41 (19.5)	29/91 (31.9)	0.022
FEV_1_/FVC < 80% (sensitivity), *n*/*N* (%)	2/31 (6.5)	18/57 (31.6)	14/41 (34.1)	48/91 (52.7)	<0.001
Bronchodilator reversibility, *n*/*N* (%)	5/31 (16.1)	16/57 (28.1)	15/41 (36.6)	43/91 (47.3)	0.008

Data are presented as *n* (%) unless otherwise indicated. *p* values were calculated using Pearson chi-square tests for categorical variables and the Kruskal–Wallis test for FEV_1_/FVC. The five prespecified clinical outcome comparisons remained statistically significant after Benjamini–Hochberg false-discovery-rate correction (all *q* ≤ 0.01). * Pulmonary function analyses were restricted to children with available spirometry data (*n* = 220). ED, emergency department; FEV_1_, forced expiratory volume in 1 s; FVC, forced vital capacity.

**Table 3:** Multivariable logistic regression analyses of associations with moderate-to-severe and uncontrolled asthma.

Variable	Moderate-to-Severe Asthma aOR (95% CI)	*p* Value	Uncontrolled Asthma aOR (95% CI)	*p* Value
**Clinical stratum**				
Familial/atopic-dominant	2.23 (0.91–5.44)	0.078	2.38 (0.88–6.43)	0.088
Wheeze-dominant	2.52 (0.99–6.37)	0.052	2.79 (1.00–7.77)	0.050
Atopic-wheezing	4.49 (1.91–10.54)	0.001	4.78 (1.85–12.35)	0.001
**Covariates**				
Age, per year	0.90 (0.77–1.06)	0.221	0.86 (0.73–1.03)	0.098
Female sex	1.04 (0.66–1.66)	0.857	0.86 (0.53–1.40)	0.554
Center 2 vs. Center 1	1.04 (0.65–1.65)	0.880	0.97 (0.60–1.58)	0.900
Passive smoke exposure	1.23 (0.76–2.00)	0.394	1.86 (1.13–3.05)	0.014
Prematurity	1.31 (0.63–2.72)	0.463	0.70 (0.31–1.59)	0.398
Eosinophilia	1.05 (0.64–1.71)	0.843	0.93 (0.56–1.55)	0.779

The low-burden stratum was the reference category. Models were adjusted simultaneously for clinical stratum, age, sex, study centre, passive smoke exposure, prematurity, and eosinophilia. The models included 14.2 and 12.0 outcome events per estimated parameter for moderate-to-severe and uncontrolled asthma, respectively. For the primary (moderate-to-severe asthma) model, discrimination was modest (apparent c-statistic 0.64; optimism-corrected 0.59 by 1000-sample bootstrap), with adequate calibration (Hosmer–Lemeshow *p* = 0.41; bootstrap-corrected calibration slope 0.70). aOR, adjusted odds ratio; CI, confidence interval.

## Data Availability

The data presented in this study are available on request from the corresponding author. The data are not publicly available owing to privacy and ethical restrictions concerning pediatric patient records.

## Supplementary Materials

The following supporting information can be downloaded at: https://www.mdpi.com/article/10.3390/jcm15166459/s1, Table S1: Clinical, wheeze-related, and atopic characteristics across predefined rule-based asthma phenotypes; Table S2: Crude and adjusted associations between age at first wheezing episode and adverse asthma outcomes; Table S3: Sensitivity and robustness analyses of the association between the atopic-wheezing phenotype and moderate-to-severe asthma; Table S4: Two-axis interaction, component-wise, and within-stratum exploratory analyses of moderate-to-severe and uncontrolled asthma; Table S5: Baseline characteristics by participating center; Table S6: Comparison of children included in vs. excluded from the spirometry and control-score analyses; Figure S1: Study flow diagram.
