# Supplementary material for: Early-Life Wheezing and Atopic Burden Are Associated with Treatment-Defined Asthma Severity and Control in School-Age Children: A Two-Center Cross-Sectional Study
Source: J Clin Med. 2026 Aug 20;15(16):6459. doi: 10.3390/jcm15166459 (PMC13513704; doi:10.3390/jcm15166459)
Supplement: Supplementary file 1 [file jcm-15-06459-s001.zip › jcm-4454145-supplementary.pdf]

Characteristic	Low-burden (n=46)	Familial/atopic-dominant (n=87)	Wheeze-dominant (n=64)	Atopic-wheezing (n=140)	p
**Demographic and clinical characteristics**					
Age, years, median (IQR)	8.8 (8.2-10.3)	9.1 (8.1-10.1)	8.9 (7.9-10.2)	9.4 (8.3-10.3)	0.525
Female sex, n (%)	21 (45.7)	38 (43.7)	25 (39.1)	61 (43.6)	0.903
Asthma diagnosis age, years, median (IQR)	6.1 (5.2-6.5)	6.5 (5.5-7.1)	5.3 (4.7-6.1)	4.7 (3.9-6.1)	<0.001
Prematurity, n (%)	4 (8.7)	8 (9.2)	9 (14.1)	15 (10.7)	0.780
Passive smoke exposure, n (%)	10 (21.7)	23 (26.4)	26 (40.6)	56 (40.0)	0.032
Bronchiolitis hospitalization, n (%)	3 (6.5)	7 (8.0)	13 (20.3)	29 (20.7)	0.014
**Wheeze-related characteristics**					
First wheeze onset at ≤ 24 months, n (%)	0 (0.0)	0 (0.0)	25 (39.1)	101 (72.1)	<0.001
High wheeze burden, n (%)	0 (0.0)	0 (0.0)	64 (100.0)	140 (100.0)	*<0.001†*
**Atopic characteristics**					
Atopic-feature count, median (IQR)	1.0 (0.0-1.0)	3.0 (3.0-4.0)	1.0 (1.0-1.0)	4.0 (3.0-4.0)	*<0.001†*
High atopic load, n (%)	0 (0.0)	87 (100.0)	0 (0.0)	140 (100.0)	*<0.001†*
Atopic dermatitis, n (%)	2 (4.3)	35 (40.2)	5 (7.8)	73 (52.1)	*<0.001†*
Food allergy, n (%)	1 (2.2)	20 (23.0)	2 (3.1)	40 (28.6)	*<0.001†*
Allergic rhinitis, n (%)	5 (10.9)	75 (86.2)	13 (20.3)	123 (87.9)	*<0.001†*
Aeroallergen sensitization, n (%)	8 (17.4)	80 (92.0)	20 (31.2)	127 (90.7)	*<0.001†*
Parental asthma, n (%)	5 (10.9)	27 (31.0)	12 (18.8)	54 (38.6)	*<0.001†*
Family atopy, n (%)	5 (10.9)	65 (74.7)	10 (15.6)	108 (77.1)	*<0.001†*
Total IgE, IU/mL, median (IQR)	64.0 (34.6-84.4)	151.7 (122-188)	52.0 (28.3-85.9)	164.4 (134-190)	<0.001
Eosinophil count, cells/uL, median (IQR)	139 (87-242)	232 (152-524)	211 (114-519)	260 (153-824)	<0.001
Eosinophilia, n (%)	6 (13.0)	27 (31.0)	19 (29.7)	65 (46.4)	<0.001

Supplementary Table S1. Clinical, wheeze-related, and atopic characteristics across predefined rule-based asthma phenotypes

Data are presented as median (interquartile range) or n (%).

p values for age, asthma diagnosis age, atopic-feature count, total IgE, and eosinophil count were obtained using the Kruskal-Wallis test. Pearson chi-square tests were used for categorical variables.

† p values marked with a dagger are shown for descriptive completeness. These differences are mathematically induced, at least in part, by the rule-based phenotype-definition algorithm and must not be interpreted as independent evidence of phenotype-associated differences.

All p values are global, exploratory, and unadjusted for multiple comparisons; no pairwise comparisons are shown in this table.

Supplementary Table S2. Crude and adjusted associations between age at first wheezing episode and adverse asthma outcomes

Age at first wheezing episode	Cases/total (%)	Crude OR (95% CI)	aOR (95% CI)	p (Wald)	p (Holm)
**Moderate-to-severe asthma** (n=337; events=128; global adjusted p=0.0038 by likelihood-ratio test)					
No early wheeze	*32/119 (26.9)*	*1.00 (Reference)*	*1.00 (Reference)*	—	—
25-72 mo	32/92 (34.8)	1.45 (0.80-2.62)	1.46 (0.80-2.66)	0.2220	0.2220
13-24 mo	31/69 (44.9)	2.22 (1.19-4.14)	**2.13 (1.12-4.04)**	**0.0209**	**0.0418**
≤12 mo	33/57 (57.9)	3.74 (1.92-7.26)	**3.31 (1.67-6.58)**	**<0.001**	**0.0019**
**Uncontrolled asthma** (n=337; events=108; global adjusted p=0.0040 by likelihood-ratio test)					
No early wheeze	*25/119 (21.0)*	*1.00 (Reference)*	*1.00 (Reference)*	—	—
25-72 mo	30/92 (32.6)	1.82 (0.98-3.38)	1.81 (0.95-3.42)	0.0705	0.0705
13-24 mo	24/69 (34.8)	2.01 (1.03-3.89)	2.22 (1.10-4.44)	0.0251	0.0501
≤12 mo	29/57 (50.9)	3.89 (1.97-7.70)	**3.60 (1.75-7.38)**	**<0.001**	**0.0014**
**≥1 exacerbation in prior 12 mo** (n=337; events=116; global adjusted p=0.0019 by likelihood-ratio test)					
No early wheeze	*29/119 (24.4)*	*1.00 (Reference)*	*1.00 (Reference)*	—	—
25-72 mo	33/92 (35.9)	1.74 (0.96-3.15)	1.71 (0.93-3.15)	0.0827	0.1655
13-24 mo	22/69 (31.9)	1.45 (0.75-2.80)	1.56 (0.79-3.07)	0.1992	0.1992
≤12 mo	32/57 (56.1)	3.97 (2.03-7.76)	**3.86 (1.92-7.78)**	**<0.001**	**<0.001**
**ACT/C-ACT <20** (n=254; events=97; global adjusted p=0.0033 by likelihood-ratio test)					
No early wheeze	*22/90 (24.4)*	*1.00 (Reference)*	*1.00 (Reference)*	—	—
25-72 mo	27/68 (39.7)	2.04 (1.03-4.03)	2.00 (0.99-4.04)	0.0531	0.0709
13-24 mo	21/50 (42.0)	2.24 (1.07-4.69)	2.29 (1.06-4.94)	0.0354	0.0709
≤12 mo	27/46 (58.7)	4.39 (2.06-9.38)	**4.23 (1.90-9.43)**	**<0.001**	**0.0012**

OR, odds ratio; aOR, adjusted odds ratio; CI, confidence interval, mo: month.
Multivariable logistic regression models used no early wheeze as the reference category.
aORs were adjusted for current age (per 1 year), sex, study center, passive smoke exposure, parental asthma, aeroallergen sensitization, and eosinophilia. All model covariates were complete. In a sensitivity analysis additionally adjusting for prematurity, estimates for all wheezing-onset categories were materially unchanged (e.g., ≤12 months: aOR 3.34, 95% CI 1.68–6.65, versus 3.31, 95% CI 1.67–6.58 in the primary model); prematurity itself was not an independent predictor (aOR 1.34, 95% CI 0.64–2.79).
p (Wald) compares each wheezing-onset category with no early wheeze. p (Holm) is the corresponding Holm-adjusted p value across the three adjusted comparisons versus the reference group within each outcome.
Global adjusted p values were obtained from likelihood-ratio tests comparing models with and without the 4-level wheezing-onset category term (3 degrees of freedom).
ACT/C-ACT <20 analyses were restricted to children with available ACT/C-ACT scores (n=254; group denominators: 90, 68, 50, and 46).
A pre-index baseline asthma-severity measure was not available. Current asthma severity, GINA step, and treatment intensity were not included in the primary models because they were assessed concurrently with, or overlap conceptually with, the outcomes and could lead to overadjustment.

Supplementary Table S3. Sensitivity and robustness analyses of the association between the atopic-wheezing phenotype and moderate-to-severe asthma

Model or exposure specification	N analysed	aOR (95% CI)	p value
**Part A. Atopic-wheezing vs low-burden contrast from models in which phenotype was entered as a four-level categorical predictor**			
Primary adjusted model	337	**4.52 (1.93-10.62)**	**<0.001**
Model excluding eosinophilia	337	4.56 (1.97-10.56)	<0.001
Additional adjustment for parental asthma†	337	4.44 (1.86-10.57)	<0.001
Excluding parent-recall early-wheeze histories	272	7.55 (2.82-20.17)	<0.001
Centre-stratified: Centre 1	182	3.97 (1.19-13.20)	0.024
Centre-stratified: Centre 2	155	5.05 (1.48-17.16)	0.010
**Part B. Alternative exposure definitions in separate binary-exposure models (each exposure vs all other children)**			
Early-onset atopic-wheezing composite‡	337	2.20 (1.34-3.59)	0.002
Stricter ≥3 atopic-feature early-onset atopic-wheezing composite§	337	2.01 (1.22-3.33)	0.006
First wheeze onset at ≤ 12 months	337	2.48 (1.37-4.49)	0.003

Outcome: moderate-to-severe asthma. Values are adjusted odds ratios (aORs) with 95% confidence intervals from logistic-regression models.

Part A primary model: phenotype was modelled as a four-level variable (low-burden reference; familial/atopic-dominant and wheeze-dominant categories retained in the model but not shown).

The primary covariate set included current age (per year), sex, study centre, passive smoke exposure, and eosinophilia. This robustness specification differs slightly from the main-text Table 3 model, which additionally adjusted for prematurity; consequently the atopic-wheezing estimate is 4.52 (95% CI 1.93–10.62) here versus 4.49 (95% CI 1.91–10.54) in Table 3. The estimated atopic-wheezing vs low-burden contrast is shown.

In centre-stratified models, study centre was omitted from the covariate set. There was no evidence that the association between phenotype and outcome varied by study centre (joint phenotype-by-centre interaction likelihood-ratio test, 3 df: P=0.635).

† Parental asthma contributes to the high-atopic-load construct used to classify the phenotypes. Therefore, this component-adjusted model is exploratory and should not be interpreted as the primary causal model.

‡ Defined as high wheeze burden plus high atopic load plus first wheeze onset at ≤ 24 months (exposure-positive n=101).

§ Defined as high wheeze burden plus >=3 atopic features plus first wheeze onset at ≤ 24 months (exposure-positive n=93).

Part B effect estimates are not directly comparable with Part A because they use separate binary exposures and a different reference group.

All analyses in this table are exploratory sensitivity analyses; p values are two-sided and nominal (not adjusted for multiple testing).

aOR, adjusted odds ratio; CI, confidence interval; df, degrees of freedom

Supplementary Table S4. Two-axis interaction, component-wise, and within-stratum exploratory analyses of moderate-to-severe and uncontrolled asthma.

Model or predictor	Adjusted OR (95% CI)	p value
**A. Two-axis model with interaction (wheeze domain × high atopic load), mirroring the phenotype definition; outcome: moderate-to-severe asthma**		
Wheeze domain (early-onset and/or high-burden)	2.56 (1.01–6.46)	0.047

Model or predictor	Adjusted OR (95% CI)	p value
High atopic load	2.24 (0.92–5.45)	0.077
Wheeze domain × high atopic load	0.80 (0.27–2.36)	0.69
**B. Individual components, each entered as a single predictor; outcome: moderate-to-severe asthma**		
Atopic dermatitis	1.64 (1.02–2.62)	0.040
Food allergy	1.34 (0.76–2.36)	0.310
Allergic rhinitis	1.60 (0.99–2.59)	0.056
Aeroallergen sensitization	1.80 (1.08–3.00)	0.025
Parental asthma	1.30 (0.80–2.12)	0.287
Family atopy	1.32 (0.84–2.07)	0.223
Early-onset wheeze (≤24 mo)	2.31 (1.45–3.69)	<0.001
High wheeze burden	2.12 (1.31–3.45)	0.002
**C. Within high atopic load (n=227): atopic-wheezing vs familial/atopic-dominant phenotype**		
Moderate-to-severe asthma	2.05 (1.15–3.66)	0.015
Uncontrolled asthma	1.98 (1.07–3.64)	0.029
**D. Parental asthma in a separate model**		
→ Moderate-to-severe asthma	1.30 (0.80–2.12)	0.287
→ Uncontrolled asthma	0.65 (0.38–1.11)	0.112

• All values are adjusted odds ratios (aORs) with 95% confidence intervals from multivariable logistic-regression models adjusted for current age (per year), sex, study centre, passive smoke exposure, and eosinophilia.

• All analyses are exploratory; p values are two-sided and nominal (not adjusted for multiple testing).

• In Part A, the wheeze domain (early-onset and/or high-burden wheeze) and high atopic load were entered simultaneously with their multiplicative interaction term, matching the two axes used to define the clinical phenotypes (phenotype variable not included). In Part B, each component was entered as the sole exposure in a separate model. Part D shows parental asthma entered separately.

Supplementary Table S5. Baseline characteristics by participating center.

Characteristic	Center 1: Şanlıurfa (n=182)	Center 2: Van (n=155)	P
**Children, n**	182	155	—
**Age, years (mean ± SD)**	9.1 ± 1.4	9.3 ± 1.4	0.078
**Male**	54.4%	60.0%	0.355
**High atopic load**	65.9%	69.0%	0.626
**Aeroallergen sensitization**	68.1%	71.6%	0.566
**High wheeze burden**	57.7%	63.9%	0.296
**Moderate-to-severe asthma**	37.4%	38.7%	0.888
**Uncontrolled asthma**	32.4%	31.6%	0.968
**Spirometry available**	65.9%	64.5%	0.875
**ACT/C-ACT available**	79.1%	71.0%	0.109

Continuous variables compared with Welch t-test; categorical variables with chi-square test. Centers were broadly comparable across characteristics.

Supplementary Table S6. Comparison of children included in vs. excluded from the spirometry and control-score analyses.

Characteristic	Spiro available (n=220)	No spiro (n=117)	P	ACT/C-ACT avail (n=254)	No ACT/C-ACT (n=83)	P
**Children, n**	220	117	—	254	83	—
**Age, years (mean ± SD)**	9.1 ± 1.4	9.4 ± 1.4	0.039	9.2 ± 1.4	9.2 ± 1.3	0.937
**Male**	56.4%	58.1%	0.846	58.3%	53.0%	0.477
**Moderate-to-severe asthma**	42.3%	29.9%	0.035	36.6%	42.2%	0.438
**Uncontrolled asthma**	35.5%	25.6%	0.086	31.1%	34.9%	0.607
**High atopic load**	67.3%	67.5%	1.000	68.1%	65.1%	0.704
**High wheeze burden**	60.0%	61.5%	0.874	60.6%	60.2%	1.000

Children with available spirometry had a higher prevalence of moderate-to-severe asthma and were marginally younger than those without; availability of ACT/C-ACT was not associated with severity. These comparisons are disclosed as a potential source of differential selection for the spirometric and control-score analyses.

Supplementary Figure S1. Study flow diagram.

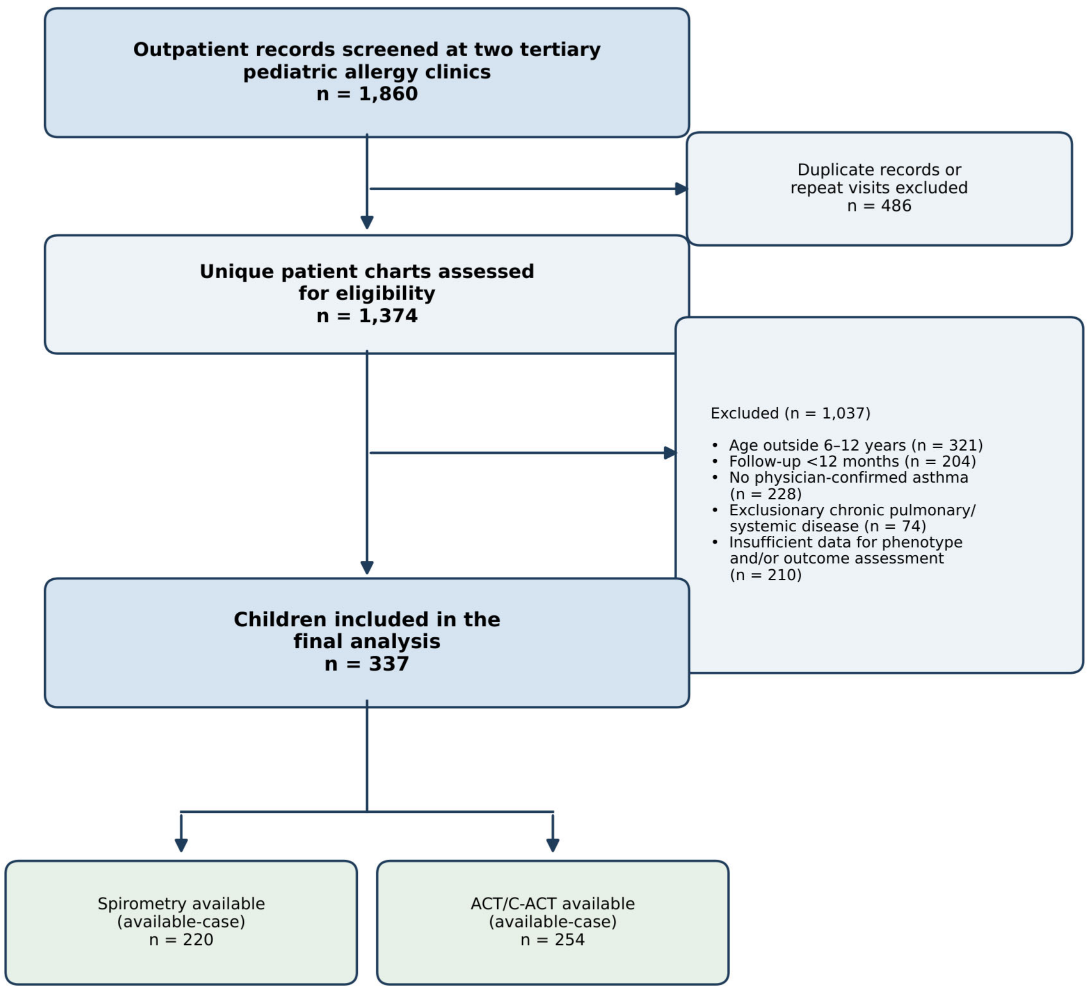

Flow diagram of record screening, eligibility assessment, and study inclusion. A total of 1,860 outpatient records from two tertiary pediatric allergy clinics were screened. After removal of duplicate or repeat-visit records (n=486) and application of the prespecified eligibility criteria, 337 children were included in the final analysis. Spirometry (n=220) and ACT/C-ACT (n=254) analyses were conducted on an available-case basis. “Insufficient data” denotes missing or unclear documentation of variables required for phenotype assignment or outcome assessment.
